# Development and Validation of a S1 Protein-Based ELISA for the Specific Detection of Antibodies against Equine Coronavirus

**DOI:** 10.3390/v11121109

**Published:** 2019-11-30

**Authors:** Shan Zhao, Constance Smits, Nancy Schuurman, Samantha Barnum, Nicola Pusterla, Frank van Kuppeveld, Berend-Jan Bosch, Kees van Maanen, Herman Egberink

**Affiliations:** 1 Virology Division, Department of Infectious Diseases & Immunology, Faculty of Veterinary Medicine, Utrecht University, Yalelaan 1, 3584CL Utrecht, The Netherlands; s.zhao@uu.nl (S.Z.); N.M.P.Schuurman@uu.nl (N.S.); f.j.m.vankuppeveld@uu.nl (F.v.K.); b.j.bosch@uu.nl (B.-J.B.); 2 GD Animal Health, Department of Small Ruminants, Horses and Companion Animals, Arnsbergstraat 7, 7418EZ Deventer, The Netherlands; c.smits@gddiergezondheid.nl; 3 Department of Medicine and Epidemiology, School of Veterinary Medicine, University of California, Davis, One Shields Ave., Davis, CA 95616, USA; smmapes@ucdavis.edu (S.B.); npusterla@ucdavis.edu (N.P.)

**Keywords:** equine coronavirus, spike S1 protein, ELISA, virus neutralization, seroprevalence

## Abstract

Equine coronavirus (ECoV) is considered to be involved in enteric diseases in foals. Recently, several outbreaks of ECoV infection have also been reported in adult horses from the USA, France and Japan. Epidemiological studies of ECoV infection are still limited, and the seroprevalence of ECoV infection in Europe is unknown. In this study, an indirect enzyme-linked immunosorbent assay (ELISA) method utilizing ECoV spike S1 protein was developed in two formats, and further validated by analyzing 27 paired serum samples (acute and convalescent sera) from horses involved in an ECoV outbreak and 1084 sera of horses with unknown ECoV exposure. Both formats showed high diagnostic accuracy compared to virus neutralization (VN) assay. Receiver-operating characteristic (ROC) analyses were performed to determine the best cut-off values for both ELISA formats, assuming a test specificity of 99%. Employing the developed ELISA method, we detected seroconversion in 70.4% of horses from an ECoV outbreak. Among the 1084 horse sera, seropositivity varied from 25.9% (young horses) to 82.8% (adult horses) in Dutch horse populations. Further, sera of Icelandic horses were included in this study and a significant number of sera (62%) were found to be positive. Overall, the results demonstrated that the ECoV S1-based ELISA has reliable diagnostic performance compared to the VN assay and is a useful assay to support seroconversion in horses involved with ECoV outbreaks and to estimate ECoV seroprevalence in populations of horses.

## 1. Introduction

Coronaviruses (CoVs) are enveloped, positive single-stranded RNA viruses that belong to the subfamily *Orthocoronavirinae* in the family *Coronaviridae* of the order Nidovirales. They are classified into four genera (*alpha-, beta-, gamma- and deltacoronavirus*) and infect both mammalian and avian hosts [1,2]. Equine coronavirus (ECoV) belongs to *Betacoronavirus 1* species, within the *Embecovirus* subgenus of the *Betacoronavirus* genus, as does human coronavirus OC43, HKU1 and bovine coronavirus [3]. ECoV was isolated for the first time from a two-week-old diarrheic foal in North Carolina (USA) in 1999, suggesting the role of ECoV in causing enteric disease [4]. Since 2010, several cases of ECoV infections have also been reported in adult horses from the United States, Europe and Japan [5,6,7,8,9]. Equine coronavirus has been detected in fecal samples from horses with clinical signs that included anorexia, lethargy, fever and, less frequently, diarrhea, colic and neurologic deficits [10,11]. The morbidity rate varies from 10% to 83% during outbreaks. Mortality is low and has been related to endotoxemia, septicemia or hyperammonemia-associated encephalopathy [12,13]. The outbreaks in adult horses demand further studies on the pathogenesis and epidemiology of ECoV infections. For this, diagnostic assays with high sensitivity and specificity are crucial.

ECoV is known to be associated with enteric infections but can also be detected in a small percentage of horses with respiratory signs. Virus shedding can be observed in fecal samples or nasal swabs from sick horses as well as healthy horses, but with a strong association between clinical signs assumed to be related to ECoV infection and virus detection in fecal samples suggesting a possible etiological role of ECoV [10,14]. Recently, real-time quantitative PCR (qPCR) methods have been established and were shown to be able to detect ECoV in feces efficiently. However, ECoV viral nucleic acid is generally only detectable by qPCR within a limited timeframe of 3–9 days post infection, as reported from both field and experimental studies [6,7,12,15]. On the other hand, serological assays can be used to support the diagnosis of a clinical ECoV infection by showing seroconversion or a significant increase in antibody titer in paired serum samples. Serological assays are also needed to gain more insight into the transmission rate of infection within animal populations [16]. Antibodies induced by betacoronaviruses persist in blood for a longer period after infection [17,18]. The virus neutralization (VN) assay has long been used as a gold standard to confirm serological responses to coronavirus infections [19,20,21]. Although the VN assay is highly specific for the detection of antibodies, it is also time-consuming and laborious to perform. Alternative high-throughput serologic assays that correlate well with neutralizing antibodies are therefore needed. Severe infections of ECoV have been shown to be associated with high viral load, but mild or asymptomatic infections may occur with low levels of virus replication being negative in PCR and with variable immune responses [12]. Consequently, specific, sensitive and high-throughput serodiagnostic methods are necessary to avoid the underestimation of prevalence in surveillance studies.

The spike protein (S) of coronaviruses is the key mediator in virus cell entry and therefore the major target for neutralizing antibodies. The S ectodomain consists of two functionally interdependent subunits, S1 and S2. The N-terminal S1 subunit is responsible for receptor binding, while the C-terminal S2 subunit mediates membrane fusion [22,23]. The S1 subunit is the most variable immunogenic antigen among coronaviruses, and therefore it is an ideal candidate for the detection of CoV species-specific antibodies [24,25]. The objective of the study was to develop and validate an ELISA method for the detection of specific antibodies to ECoV and provide a tool for the diagnosis and the future estimation of ECoV prevalence and incidence in various equine (sub) populations.

## 2. Materials and Methods

### 2.1. Equine Serum Panels

A total of 1138 equine serum samples were included in this study. The details of serum panels A–H (*n* = 1084) are shown in Table 1. They were retrieved from the serum bank at GD Animal health Deventer, the Netherlands. All of them were collected for the monitoring of other diseases independent to this study, and their ECoV exposure status was unknown. With the exception of panel H (collected from Iceland), all serum samples from panel A to G were collected from horses in the Netherlands. Additionally, panel I included 27 paired (acute- and convalescent-phase) serum samples that were collected during an ECoV outbreak in the USA (2014). All samples were stored at −20 °C until tested.

### 2.2. Cells and Virus

ECoV strain NC99 was propagated and titrated in human rectal adenocarcinoma (HRT-18G) cells. HRT-18G cells and human embryonic kidney 293 cells stably expressing the SV40 large T antigen (HEK-293T) were maintained in Dulbecco modified Eagle medium (DMEM, Lonza, Basel, Switzerland) containing glutamine and supplemented with 10% fetal bovine serum (FBS, Bodinco, Alkmaar, The Netherlands), penicillin (100 IU/mL), and streptomycin (100 µg/mL).

The ECoV NC99 and HRT-18G were obtained from Dr. Udeni B.R. Balasuriya, School of Veterinary Medicine, Louisiana State University, USA [3,4].

### 2.3. Plasmids Design and Protein Expression

The sequence of the S1 subunit of the spike protein of the ECoV NC99 strain (residue 1–762 of the amino acid sequence) was derived from Genbank (Genbank No.: EF446615.1). Human codon-optimized sequences encoding the ECoV S1 subunit were synthesized and fused to the Fc domain of mouse IgG2a, which was subsequently cloned into the pCAGGS mammalian expression vector as described before [26]. For ECoV S1-Fc protein production, expression plasmid was transfected into HEK-293T cells using polyethyleneimine (Polysciences, Inc., Warrington, PA, USA) in a ratio of 1:10. After 6 h of incubation, the transfection medium was removed and replaced by 293 SFM II expression medium (Gibco^®^, Life Technologies Inc., Grand Island, NY, USA). At six days post transfection, cell culture supernatants were harvested and the soluble S1 was purified from the culture medium using Protein A Sepharose beads (GE Healthcare Bio-Sciences AB, Uppsala, Sweden). Subsequently, the proteins were eluted using 0.1M citric acid, pH 3.0, and immediately neutralized with 1 M Tris-HCl, pH 8.8. The purity and integrity of proteins were analyzed by sodium dodecyl sulphate polyacrylamide gel electrophoresis (SDS-PAGE) and stained with GelCodeBlue stain reagent (ThermoFisher Scientific Inc., Waltham, MA, USA). Purified proteins were quantified by Nanodrop spectrophotometry (ThermoFisher Scientific Inc., Waltham, MA, USA) and by sodium dodecyl sulphate polyacrylamide gel electrophoresis (SDS-PAGE) with bovine serum albumin (BSA) as standard, then stored at −80 °C until further usage.

### 2.4. Virus Neutralization (VN) Assay

Equine sera (*n* = 231) were randomly selected from different serum panels (A–D) and tested for neutralizing antibody titers in an ECoV VN assay. Heat-inactivated equine sera (56 °C for 30 min) were serially diluted 2-fold in DMEM supplemented with 2% fetal bovine serum and mixed with an equal volume of ECoV NC99 strain (100 50% tissue culture infective doses (TCID_50_)/well) in 96-well cell culture plates (Corning Inc., Kennebunk, ME, USA). Virus–serum mixtures were incubated at 37 °C for 60 min. Then 100 µL of the virus–serum mixture was added in duplicate to HRT-18G cells monolayers in 96-well cell culture plates. At six days post infection, a clear cytopathic effect (CPE) was observed and the virus neutralization titers (VNT) were determined. The VNT of sera were expressed as the reciprocals of the highest serum dilution that resulted in 90% neutralization of CPE. A titer of ≥8 was considered to be positive.

### 2.5. ECoV S1 ELISA Development

Two different formats were developed employing ECoV S1 protein, a so-called wet format ELISA (wELISA) and a dry format ELISA (dELISA).

#### 2.5.1. ECoV S1 Wet Format ELISA (wELISA)

High-binding microtiter plates (Greiner Bio-one BV, Alphen aan den Rijn, The Netherlands) were coated with ECoV S1 protein (100 µL per well) in phosphate buffered saline (PBS, pH 7.4) overnight at 4 °C. The optimal protein amount and dilution of secondary antibody conjugate were determined by checkerboard titration. The protein concentration in use was 0.25 µg/mL. After three washes with PBS containing 0.05% Tween-20 (PBST), the plates were blocked with PBST containing 5% milk powder (Protifar, Nutricia, Zoetermeer, The Netherlands) for 2 h at 37 °C. Following blocking, plates were incubated with serum samples diluted 1:200 in PBST containing 5% milk powder for 1 h at 37 °C. After a washing step, 100 µL/well 1:20,000 diluted horseradish peroxidase (HRP)-conjugated goat anti-horse IgG (H&L) (Abnova, Taiwan, China) was added to detect bound antibodies and plates were incubated for 1 h at 37 °C. Subsequently, the plates were washed, and the peroxidase reaction was then visualized via incubating plated with TMB Super Slow One Component HRP Microwell Substrate (BioFX^®^, Surmodics IVD, Inc., Eden Prairie, MN, USA) for 10 min at room temperature. The reaction was stopped by adding 12.5% sulfuric acid (H_2_SO_4_ (VWR International BV, Amsterdam, The Netherlands)) and optical densities (OD) were immediately measured at 450 nm using an ELISA microplate reader (BioTek Instruments, Inc., Winooski, VT, USA). All serum samples were tested in duplicate.

#### 2.5.2. ECoV S1 Dry Format ELISA (dELISA)

High-binding microtiter plates (Greiner Bio-one BV, Alphen aan den Rijn, The Netherlands) were coated with ECoV S1 protein (100 µL per well) in ammoniumcarbonate solution (9.8 g/L (VWR International BV, Amsterdam, The Netherlands)) overnight at 4 °C. Then 100 µL/well blocking solution (9.8 g/L ammoniumcarbonate + 4 g/L caseine (VWR International BV, Amsterdam, The Netherlands) + 20 g/L sucrose (Merck and Co., Inc., Kenilworth, NJ, USA)) was added and plates were incubated for one hour at room temperature. Subsequently, the contents of the plates were discarded, and plates were dried for four hours at 37 °C, vacuum sealed and stored at 4–8 °C. The optimal protein amount and dilution of secondary antibody conjugate were determined by checkerboard titration. The protein concentration in use was 0.13 µg/mL. Plates were incubated with serum samples 100 µL per well and diluted 1:200 in PBS + 0.05% Tween-20 + 2.5% dry milk (Bio-Rad Laboratories, Inc., Hercules, CA, USA) for 1 h at 37 °C. After a washing step (five times with PBST 300 µL/well on a Biotek automatic washing station), 100 µL per well 1:60,000 diluted horseradish peroxidase (HRP)-conjugated goat anti-horse IgG (H&L) (Abnova, Taiwan, China) was added to detect bound antibodies and incubated for 1 h at 37 °C. Subsequently, the plates were washed again using the same washing procedure and the peroxidase reaction was then visualized by incubating plates with TMB (IDEXX Laboratories, Westbrook, NJ, USA) for 15 min at room temperature. The reaction was stopped by adding 50 µL/well sulfuric acid (H_2_SO_4_ 0.5 M (VWR International BV, Amsterdam, The Netherlands)) and optical densities (OD) were immediately measured at 450 nm using an ELISA microplate reader (BioTek Instruments, Inc., Winooski, VT, USA). All serum samples were tested in duplicate. S/P values were calculated with the formula: S/P = (OD Sample-OD Negative control)/(OD Positive control-OD Negative control).

### 2.6. Statistical Analysis

The correlation between OD values scored with two ELISA formats was measured by the Pearson correlation coefficient using Graph Pad Prism, version 7. The discriminating power of the two different ELISA formats was analyzed by performing receiver operator characteristic (ROC) analysis with 231 sera, which 94 were negative (VNT < 8) and 137 were positive (VNT ≥ 8). The cut-off value, diagnostic specificity and sensitivity were determined by ROC analysis using Sigmaplot. A minimum specificity of 99% was chosen for the selection of cut-off values. Additionally, the reproducibility of assays was evaluated by testing three samples with different OD values. Inter-assay coefficients of variation (CV) and intra-assay CV were determined testing each sample in triplicate on three different plates in three different runs and within the same plate, respectively.

## 3. Results

### 3.1. Determination of Neutralizing Antibodies

To identify equine sera containing ECoV-neutralizing antibodies, we screened a subset of 231 equine sera, composed of randomly selected serum samples from panels A–C, and all samples from panel D were screened in the VN assay. Of the 231 sera, 94 sera were tested as negative (titers < 8) and 137 positive samples (titers ranging from 8 to 4096).

Additionally, paired samples from 27 horses (*n* = 54, panel I) were tested in the VN assay. Twenty out of 27 sera collected from the first time point exhibit titers ranging from 12 to 2048. The convalescent serum samples were collected 21–28 days following the first round of sample collections, and all of them showed neutralization responses with titers ranging from 16 to 4096. Within these horses, seven of them showed seroconversion and 14 showed a significant (4-fold or greater: 2log2) increase in titer in the VN assay. To confirm the presence of ECoV specific IgG in Icelandic horses, 24 horse sera with positive ECoV S1 ELISA results (in panel H, S/P value > 0.5) were tested in ECoV neutralization assays. All of them had neutralizing antibodies with titers varying between 32 and 768.

### 3.2. Development of ECoV S1 ELISA

**Figure 1:**
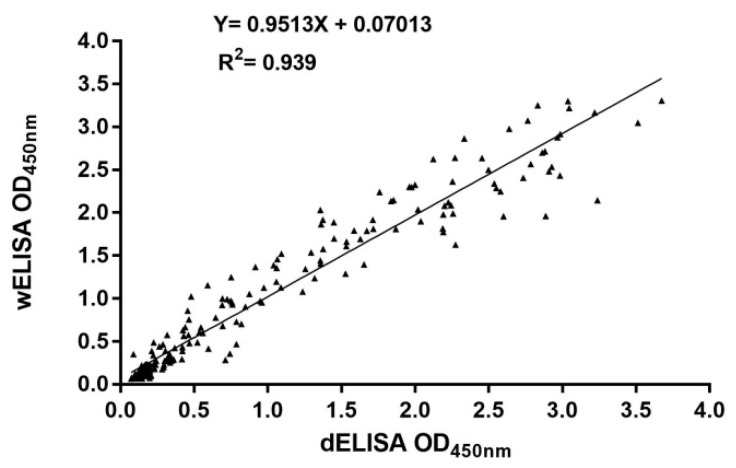
Correlation between optical density (OD) values obtained with wet format ELISA (wELISA) and dry format ELISA (dELISA).

Besides the conventional wet ELISA format (wELISA) for general laboratory usage, a dry standardized ELISA format (dELISA) was also developed and validated to facilitate implementation as routine diagnostic method in different laboratories and possibly wider application as an ELISA kit. Both ELISA formats were developed for the detection of ECoV-specific antibodies in horse serum samples. The diagnostic performance of both ELISAs was evaluated using a subset of 231 horse sera with known VN results as described above. The Pearson correlation coefficient was calculated to assess the correlation between the OD values obtained with the two ELISA formats (Figure 1). Results indicate that OD values obtained with both ELISAs show a high degree of correlation, with correlation and regression coefficients close to 1 (R^2^ = 0.939, regression coefficient = 0.9513, *p* < 0.0001). Thus, the performance of both ELISA formats is very similar.

**Figure 2:**
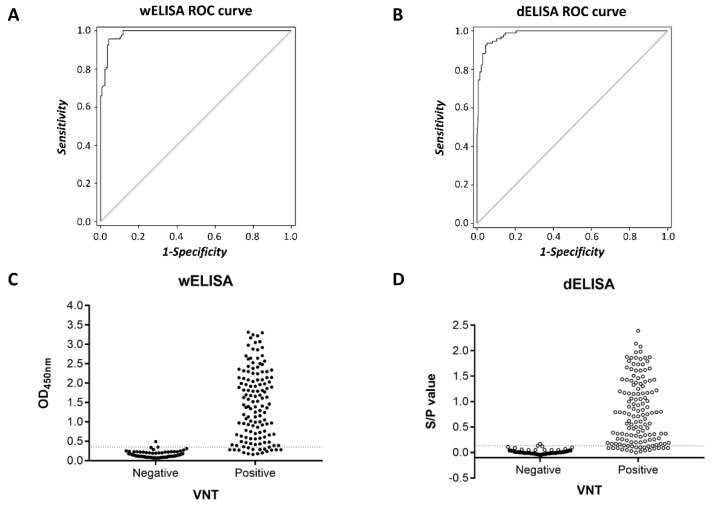
Receiver operating characteristic (ROC) analyses of equine coronavirus (ECoV) S1 ELISAs. ROC curves for wELISA (**A**) and dELISA (**B**) were plotted with positive (*n* = 137) and negative (*n* = 94) sera confirmed via VN assays. The area under the curve (AUC) is 0.985 for both ELISA formats. Distributions of wELISA (**C**) and dELISA (**D**) with confirmed sera are shown above. Calculated cut-off points are indicated by the vertical dashed lines. VN assays, virus neutralization assays; VNT, virus neutralization titer.

Subsequently, the discriminating power of the wELISA and dELISA was evaluated via receiver operator characteristic (ROC) analysis. The ROC curves were plotted based on the previous classification of 231 sera into negative and positive by VN assays (Figure 2A,B). Then the optimal cut-off values, diagnostic specificity and sensitivity of both ELISA formats were determined by the established ROC curves. The ELISA results of the VNT-positive and negative samples are shown in Figure 2C,D. The diagnostic accuracy of both ELISA formats was considered to be high as the same area under the curve (AUC) values were observed (AUC = 0.985), with a relative sensitivity and specificity approximately 95% according to the Youden plot of wELISA and dELISA. Therefore, the test characteristics of both ELISA formats were assigned the same weight. In this study, a minimum specificity of 99% was chosen for the threshold of cut-off values for both ELISAs. Accordingly, the optimal cut-off for wELISA was an OD value of 0.35—for which, the sensitivity was 87% and the specificity was 99%. For dELISA, the test results were expressed as S/P values. A cut-off at an S/P value of 0.13 yielded a sensitivity of 85% and specificity of 99%, respectively.

Furthermore, the inter- and intra-coefficient of variation (CV) of the three ECoV positive sera tested with both ELISA formats were lower than 12%. More specifically, the intra-assay CV of wELISA and dELISA ranged from 3.04% to 4.87% and from 5.4% to 7.7%, respectively, while the inter-assay CV of wELISA and dELISA varied from 4.9% to 10.26% and from 8.9% to 11.2%, respectively. Overall, these results indicate that the performances of both ELISA formats were very much equivalent and that the results of both ELISAs were strongly correlated to VN results.

### 3.3. Detection of Antibodies against ECoV in Horses during an Acute Outbreak

**Figure 3:**
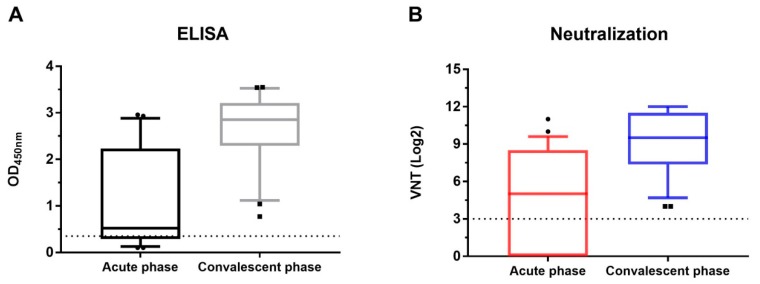
Antibodies response against ECoV from 27 horses during an acute outbreak. Boxplots show the ELISA reactivities (**A**) and VNT (**B**) of 27 horses from acute and convalescent-phase sera. Each cut-off is indicated by the dotted dashed line; VNT, virus neutralization titer.

To determine the diagnostic performance of the ECoV S1 ELISA, 27 paired serum samples (panel I) collected from an acute ECoV outbreak were investigated by wELISA. The horses presented similar clinical signs as described in [27], and virus shedding was confirmed by qPCR analysis [7]. At the acute stage, 11 out of 27 horses were qPCR positive, while at the convalescent stage, this number had decreased to six. Serum samples were further validated by VN assay (Figure 3B; Table S1). Seven out of 27 horses showed seroconversion, while another 14 horses showed a significant (4-fold or greater) increase in VNT. Performing the wELISA (see Figure 3A; Table S1), the same seven out of 27 horses showed seroconversion; acute phase sera were negative (OD value < 0.35) whereas the convalescent phase sera all had OD values greater than 1.00 (1.14–2.90). Thus, seroconversion rates calculated from wELISA and VNT showed a 100% correlation (Table S1). For the horses that showed a 4-fold or greater increase in VNT (*n* = 14), nine of the acute phase sera had positive OD values between 0.35 and 0.70 (2x background) and also a higher than 2 (*n* = 2) to 4 (*n* = 7) fold increase in the OD value in the convalescent serum. Five of the VNT positive paired serum samples had OD values of >0.70 (twice the background OD value) in the acute phase serum. Two of these samples with an OD value of 1.12 and 1.41 respectively in wELISA also showed a greater than 2-fold increase in OD value. The three VNT positive samples with less than 2-fold increase in OD values already had high OD values in the acute phase serum as well as high VNT (mean OD value = 2.51, mean VNT = 8.30). For the six horses that did not show a significant rise in VNT, five serum samples collected at the acute stage already had high antibody levels as shown by ELISA and neutralization assay (mean OD value > 2.6, mean VNT > 9, Table S1). Further, the Pearson correlation coefficient was calculated to assess the overall correlation between the OD values obtained with wELISA and VNT (log2 titers) from acute and convalescent-phase sera of the 27 horses (Figure S1). Results indicate that OD values and VNT show a good degree of correlation (R^2^ = 0.83, *p* < 0.0001). These data support the use of the wELISA as a diagnostic tool in case of suspected ECoV outbreaks.

### 3.4. ECoV Seroprevalence in Horses with Unknown ECoV Exposure

We further set out to determine the seroprevalence in horses with unknown ECoV exposure using the dELISA format. A total of 1084 serum samples (Table 1, panel A–H) were analyzed. With the exception of panel D, all sera were from adult horses (older than 36 months). Seroprevalence varied from 25.9% (panel D) to 82.8% (panel C) among these eight serum panels. The lowest number of positive samples was found in panel D which contained young horses (6-30 months old, average age: 8.38 months (95% CI 6.975–9.785)). In the other four serum panels (panel A, B, E and F) from Dutch horses, the historical serum samples (panel G) and samples from Iceland (panel H) higher seroprevalences were found (59.2–82.8%).

## 4. Discussion

Since the beginning of the 21st century, ECoV infections have been reported in horses, causing fever and enteric diseases [4]. More recently, infections in adult horses were reported with clinical signs of fever, anorexia, lethargy and, less commonly, specific signs of diarrhea and colic [7,8]. Nevertheless, information regarding the circulation of ECoV in the equine population, especially in Europe, is still limited [6,28]. Serological studies are useful tools to investigate ECoV prevalence in horse populations. In the present study, our aim was to develop a simple and reliable method for antibody detection against ECoV that can be used for diagnostics and sero-epidemiological studies.

As compared to virus neutralization assays, the ELISA method has the advantage of being reproducible, potentially high-throughput and much less laborious. In our study, we set up an ECoV S1-based ELISA method in two complementary formats. The conventional wELISA format is for general laboratory usage with simplified, easy to perform coating procedures. On the other hand, coated plates of the dELISA format could be stored for a longer time period, making it ideal for transportation and kit development. We showed that both formats performed equally well, and their results correlated nicely. When comparing with the VN assay by ROC analysis, our ELISA method with both formats was shown to have high accuracy. In our current study we applied wELISA for the analysis of the paired outbreak samples, while the dELISA was further validated and used for the high-throughput screening of larger amount of serum samples.

We utilize ECoV S1 as the viral antigen for antibody detection in this study. The S1 chimeric protein was expressed in mammalian cells, and hence both the protein conformation and modification (e.g., glycosylation) are mimicking the S proteins on the surface of virus particles [29]. As the most divergent and immunodominant component of coronaviruses, S1 has been widely used in the development of methods for specific coronavirus serological studies [19,20,26,30]. Our findings validate that ECoV S1 is a highly suitable antigen for the detection of antibodies against ECoV showing very good agreement between the ELISA and VN assays. Recently, similar conclusions were also drawn for the role of MERS S1 in MERS serology [31].

With our wELISA method, we were able to analyze paired samples that were collected during an ECoV outbreak. In the virus neutralization assay seroconversion or a 4-fold or greater increase in ECoV antibody titers could be detected in sera of 21 out of 27 horses within weeks of the initial observation of clinical disease and detection of viral RNA in feces. Of these 21 positive horses 18 showed seroconversion or a 2-fold or higher increase in OD values in the wELISA. The three remaining VNT positive samples had high OD values already in the acute phase serum. Of the six ECoV negative paired samples five had high VN antibody titers and OD values already at the acute phase. This might be due to late sampling of these horses or previous exposure to ECoV (Table S1). This study confirms that the ECoV S1 ELISA is a useful diagnostic test for the demonstration of a potential ECoV outbreak and should be considered as a useful adjunct to investigation of fecal samples by qPCR.

We also determined the seroprevalence of serum samples collected from horses with unknown ECoV exposure via our dELISA. Results showed that the overall seroprevalence in the different cohorts tested is 25.9%–82.8%. These percentages are in agreement with the study performed by Hemida et al. [32], in which they detected coronavirus infections in horses in Saudi Arabia and Oman and they found that 74% of them had detectable neutralizing antibodies to ECoV. A lower percentage (9.6%) of positive animals was found in another ECoV seroprevalence study conducted in the USA [33]. Several factors might contribute to these differences in results. There is only limited information regarding ECoV prevalence in Europe including the Netherlands [6,28], and it is possible that the overall ECoV distribution differs between continents. Moreover, our study employs eukaryotically expressed ECoV S1 protein as coating antigens, while in the US study chimeric S2 protein expressed in Escherichia coli was used. The expression in mammalian cells guarantees a more native configuration of the protein, in particular of glycosylated antigens such as the coronavirus spike protein. Reports had shown that both coronavirus S1 and S2 subunit elicit antibody responses, but the level of immune responses triggered by them may differ [34,35]. Furthermore, the criteria for determining the cut-off value are different for the two studies. In our study we defined positive and negative samples on the basis of a VN assay, whereas the US study used negative qRT-PCR and absence of clinical signs as criteria to define horses as ECoV negative. In this way, seropositive horses may have contributed to higher cut-off values and potentially a lower sensitivity of the assay.

In our study, we noticed differences in seroprevalence between young and adult horses. In the group of young horses (panel D, Table 1), the lowest seroprevalence was found. Young horses may initially be protected against ECoV infection by maternal antibodies and may become gradually more susceptible as maternal antibodies wane. The risk of becoming infected increases with age. This hypothesis is further supported by the age distribution of PCR-confirmed ECoV infection cases: foals (age 0–6 months) have the lowest infection rates, and the infection rate increases with age [10].

We also observed a significant percentage of seropositive horse serum samples collected back in 1990 (panel G, Table 1). ECoV-like viruses were detected in the 70s and 80s by electron microscopy in feces of horses with enteric disease, but virus isolation and characterization was not reported [36,37,38,39]. The history of ECoV presence, especially in Europe, is possibly much longer than currently understood [6]. Intriguingly, we noticed that Icelandic horses also are seropositive against ECoV (panel H, Table 1). Twenty-four serum samples showed high ECoV ELISA reactivity (S/P value > 0.5) and also had neutralizing antibodies with VNT varying between 32 and 768. The horse population of Iceland has been geographically isolated for more than 1000 years and is free from most common equine contagious diseases such as equine influenza, equine herpesvirus 1, strangles and equine viral arteritis [40]. To date, no prior studies of ECoV prevalence in horses from Iceland had been performed. This is the first evidence of the existence of ECoV infection in Iceland.

In conclusion, we developed a high-throughput, reliable and specific ELISA method to study humoral immune responses in horses against ECoV. With this method, we are able to perform the serodiagnosis of ECoV infection and assess the seroprevalence within horse populations in the future.

## Figures and Tables

**Table 1:** Prevalence of ECoV S1-reactive antibodies in equine sera used in this study.

Panel	Samples Source/Project Names	Collection Year	Country	Numbers of Samples	Numbers of ECoV-S1 Positive Samples	Seroprevalence (%)
A	West Nile virus (WNV) surveillance	2016	The Netherlands	167	128	76.60
B	Equine infectious anemia (EIA) surveillance	2016	The Netherlands	112	80	71.40
C	Export horses	2016	The Netherlands	99	82	82.80
D	Influenza surveillance	2015	The Netherlands	81	21	25.90
E	WNV surveillance	2015	The Netherlands	176	145	82.40
F	EIA surveillance	2015	The Netherlands	184	109	59.20
G	Equine herpesvirus 1 and 4 diagnostic serum panel	1990	The Netherlands	165	93	56.40
H	Horse sera from Iceland	2018	Iceland	100	62	62.00

## Supplementary Materials

The following are available online at https://www.mdpi.com/1999-4915/11/12/1109/s1. Table S1: Detection of antibodies to ECoV in equine serum samples during an ECoV outbreak by wELISA in winter 2014; Figure S1. Correlation between the OD values obtained with wELISA and virus neutralization titers (VNT) of 27 horses from acute and convalescent-phase sera.
