# Supplementary material for: Development and Validation of a S1 Protein-Based ELISA for the Specific Detection of Antibodies against Equine Coronavirus
Source: Viruses. 2019 Nov 30;11(12):1109. doi: 10.3390/v11121109 (PMC6950238; doi:10.3390/v11121109)
Supplement: Supplementary file 1 [file viruses-11-01109-s001.zip › Supplementary Materials/Figure S1.docx]

**Supplementary Figure**


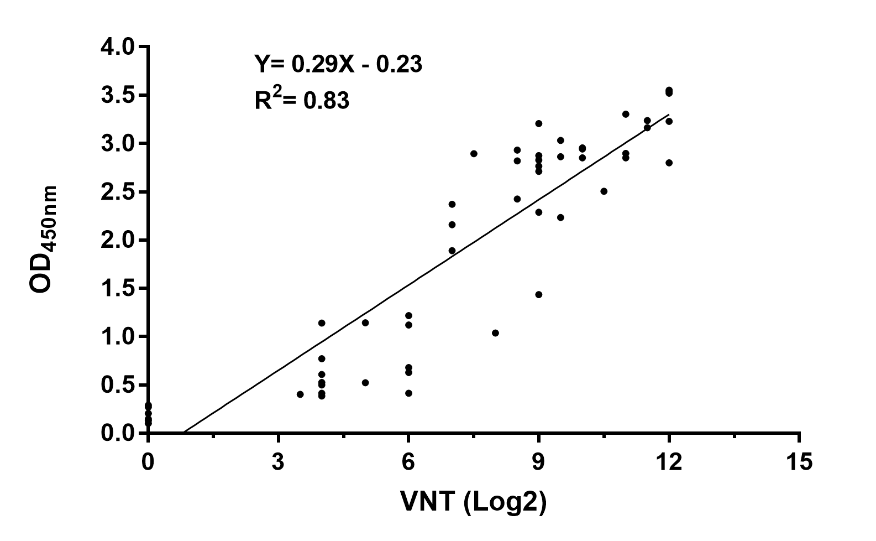


**Figure S1**. Correlation between the OD values obtained with wELISA and virus neutralization titers (VNT) of 27 horses from acute and convalescent-phase sera. Negative sera [VNT <8, (3log2)], are represented as 0 in the graph.
