# Supplementary figures and images for: Development and Validation of a S1 Protein-Based ELISA for the Specific Detection of Antibodies against Equine Coronavirus
Source: Viruses. 2019 Nov 30;11(12):1109. doi: 10.3390/v11121109 (PMC6950238; doi:10.3390/v11121109)

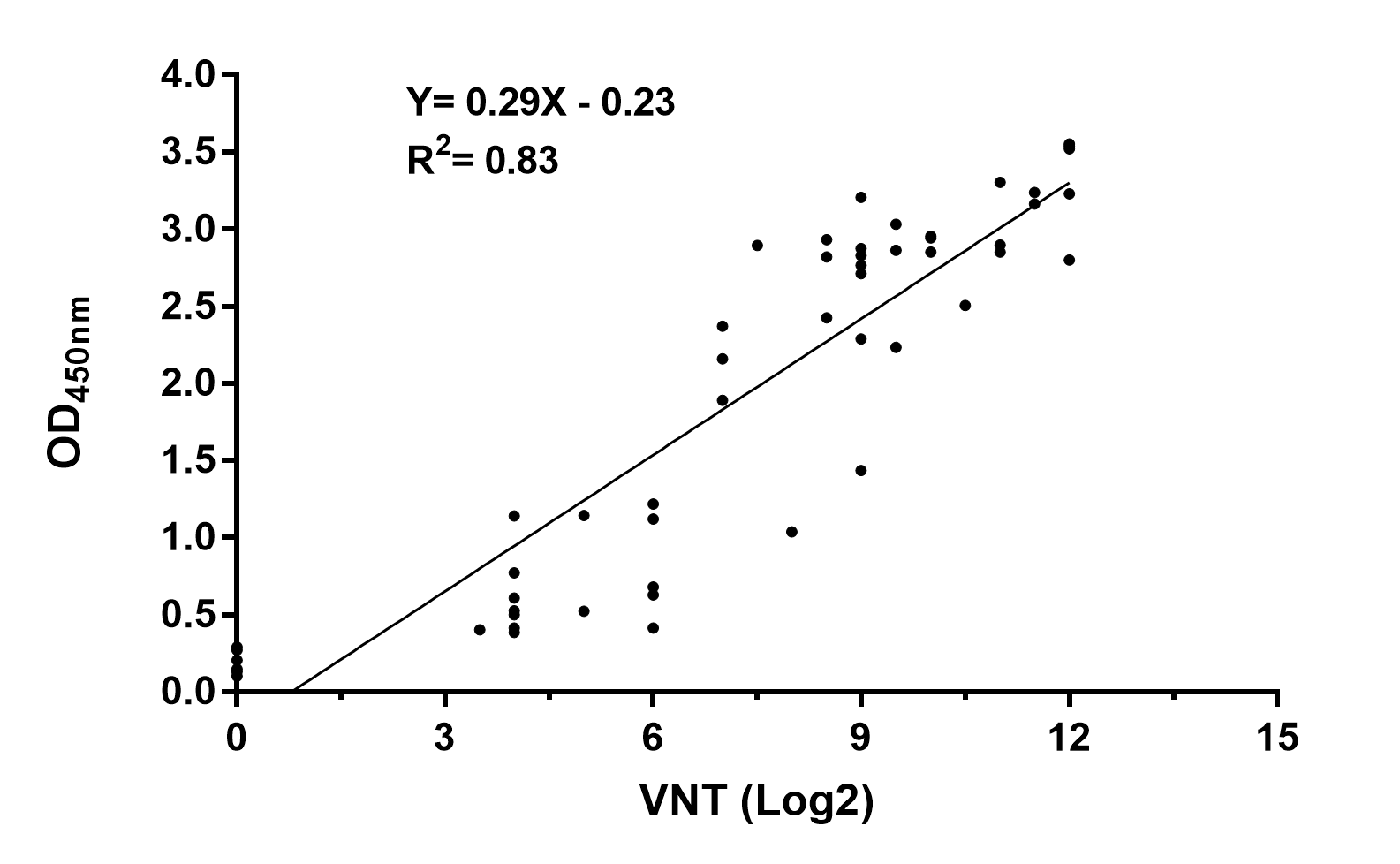

Supplement: Supplementary file 1 [file viruses-11-01109-s001.zip › Supplementary Materials/Figure S1.tif]
